# Photoelectrochemical CO_2_ Reduction Products Over Sandwiched Hybrid Ga_2_O_3_:ZnO/Indium/ZnO Nanorods

**DOI:** 10.3389/fchem.2022.814766

**Published:** 2022-02-09

**Authors:** Hye Ji Jang, Ju Hyun Yang, Ju Young Maeng, Min Hee Joo, Young Jun Kim, Choong Kyun Rhee, Youngku Sohn

**Affiliations:** ^1^ Department of Chemistry, Chungnam National University, Daejeon, South Korea; ^2^ Department of Chemical Engineering and Applied Chemistry, Chungnam National University, Daejeon, South Korea

**Keywords:** ZnO nanorod, electrochemical CO_2_ reduction, indium, Ga_2_O_3_, sandwiched hybrid

## Abstract

Recycled valuable energy production by the electrochemical CO_2_ reduction method has explosively researched using countless amounts of developed electrocatalysts. Herein, we have developed hybrid sandwiched Ga_2_O_3_:ZnO/indium/ZnO nanorods (GZO/In/ZnO_NR_) and tested their photoelectrocatalytic CO_2_ reduction performances. Gas chromatography and nuclear magnetic spectroscopy were employed to examine gas and liquid CO_2_ reduction products, respectively. Major products were observed to be CO, H_2_, and formate whose Faradaic efficiencies were highly dependent on the relative amounts of overlayer GZO and In spacer, as well as applied potential and light irradiation. Overall, the present study provides a new strategy of controlling CO_2_ reduction products by developing a sandwiched hybrid catalyst system for energy and environment.

## 1 Introduction

Producing recycled energy products using abundant CO_2_ is another challenging project for future solutions for energy and environment (Sohn et al., 2017; Wang et al., 2019; Prabhu et al., 2020; Song et al., 2020; Bellardita et al., 2021; Chen et al., 2021; da Silva Freitas et al., 2021; Ješić et al., 2021; Ochedi et al., 2021; Sun et al., 2021). Electrochemical CO_2_ reduction is a promising method, and the development of electrodes is a major goal for approaching practical application in the industry. Among many materials such as pure metals and metal oxides, ZnO, indium (In), and Ga_2_O_3_ show very unique CO_2_ reduction products after electrochemical reaction (Sekimoto et al., 2016; Tatsumi et al., 2017; Kikkawa et al., 2018; Lu et al., 2018; Qin et al., 2018; Yamamoto et al., 2018; Zhang et al., 2018; Liu et al., 2019; Ma et al., 2019; Murali et al., 2019; Zhang et al., 2019; Akatsuka et al., 2020; Daiyan et al., 2020; Hou et al., 2020; Luo et al., 2020; Tan et al., 2020; Yang et al., 2020; Deng et al., 2021; Li et al., 2021; Liang et al., 2021; Ma et al., 2021; Teng et al., 2021; Wang et al., 2021; Wei et al., 2021; Wu et al., 2021; Xiao et al., 2021; Yoon et al., 2021).

ZnO (and metallic Zn) is known to predominantly produce CO with a high Faradaic efficiency by electrochemical CO_2_ reduction (Lu et al., 2018; Qin et al., 2018; Zhang et al., 2018; Liu et al., 2019; Daiyan et al., 2020; Luo et al., 2020; Tan et al., 2020; Deng et al., 2021; Wang et al., 2021). Thereby, Zn-based materials have been chosen for CO and syngas (CO and H_2_) production. Because of optical band gaps near 400 nm, it has been used extensively as good photo(electro)catalysts (Ansari et al., 2013; Choi et al., 2015; Kang et al., 2019; Yu et al., 2020). For metallic In, it has been used for formate (or CO) production by the electrochemical method (Ma et al., 2019; Murali et al., 2019; Zhang et al., 2019; Hou et al., 2020; Yang et al., 2020; Li et al., 2021; Ma et al., 2021; Teng et al., 2021; Wei et al., 2021; Wu et al., 2021; Xiao et al., 2021), where two major reaction channels include 1) forming surface CO (from HOOC_ad_) and 2) surface OCHO (Li et al., 2021). Desirable CO_2_ reduction products have been obtained by modification of In surface that include hybridization (metal/metal and metal-nonmetal), defects, core-shells, and synthetic methods (Ma et al., 2019; Murali et al., 2019; Zhang et al., 2019; Hou et al., 2020; Yang et al., 2020; Li et al., 2021; Ma et al., 2021; Teng et al., 2021; Wei et al., 2021; Wu et al., 2021; Xiao et al., 2021). For Ga_2_O_3_ as a catalyst for CO_2_ reduction, CO and H_2_ productions are competitively occurring and dependent on the crystallinity (Akatsuka et al., 2020; Yoon et al., 2021).

Motivated by the current electrocatalysts development, herein sandwiched hybrid GZO/In/ZnO_NR_ electrodes were developed, and their photoelectrochemical CO_2_ reduction activities were examined. ZnO was chosen as a support and metallic In was used as a spacer material. GZO was used as the topmost layer and sputter-deposited to obtain a hybrid property of both Ga_2_O_3_ and ZnO. GZO (Ga_2_O_3_:ZnO) is known to be a good candidate material for transparent conducting electrode (Beckford et al., 2021). For this reason, it was first introduced as an electrode material in electrochemical CO_2_ reduction if there were any new performances. Moreover, we examined if these sandwiched hybrid materials showed any differences, compared with those for ZnO support, In/ZnO, and GZO/ZnO electrode materials.

The novelty of this study was to show the roles of spacer and the topmost layers in the developed sandwiched electrodes. Thereby, the present study provides new strategic information on the development of sandwiched hybrid electrodes for energy and environment.

## 2 Experimental Section

### 2.1 Preparation of ZnO_NR_ and Sandwiched GZO/In/ZnO_NR_ Electrodes

ZnO nanorods (ZnO_NR_) were directly grown on a Zn plate (99.9%, 2 mm thick) to use as an electrode support. For the ZnO_NR_ support, a Zn plate (5 mm × 30 mm) was dipped in a mixed solution of 50 ml deionized water and 1 ml of NH_4_OH (30%) solution. The Zn plate-dipped solution in a Teflon-lined (100 ml) autoclave was tightly capped and placed in an oven. The oven temperature was slowly increased to 120°C and then kept for 12 h. After the reaction time and natural cooling to room temperature, the autoclave reactor was opened, and then, the plate was removed, washed with deionized water, and dried under an infrared lamp.

For the preparation of an In/ZnO_NR_ electrode ***,*** indium was sputter-coated on an as-prepared ZnO_NR_ plate (5 mm × 30 mm) using an indium sputtering target (99.99%, Made Lab Co.) and an SPT-20 ion sputter coater (COXEM Co., Korea). The experimental conditions were an ionization current of 3 mA and deposition times of 60, 120, 240, 480, and 960 s. For the preparation of a GZO/In/ZnO_NR_ electrode, GZO (99.99%, Ga_2_O_3_:ZnO = 7:3 at % ratio, Made Lab Co.) was sputter-coated on an In/ZnO_NR_ electrode described above. For GZO sputtering, the experimental conditions were an ionization current of 5 mA and deposition times of 10, 100, and 800 s. The consequent sandwiched electrode was abbreviated as GZO(deposition time)/In(deposition time)/ZnO_NR_, for example, GZO(960s)/In(240s)/ZnO_NR_.

### 2.2 Characterization of Electrodes

The crystal phases of prepared electrode samples were examined using an X-ray diffractometer (Rigaku MiniFlex II) with a Cu K_α_ X-ray radiation (CNU Chemistry Core Facility). The surface morphology was examined using a scanning electron microscope (SEM, Hitachi S-4800) at a 10.0 keV condition. Energy-dispersive X-ray spectroscopy (EDXS) was employed to examine elemental compositions and mapping images using an SEM (Merlin Compact, Carl Zeiss, Germany) coupled with an AZtec Energy X-MaxN EDXS (OXFORD, Oxford, United Kingdom). UV–visible (UV-Vis) absorption property was examined using a double beam UV–visible spectrophotometer (SCINCO NeoSys-2000) with a diffuse reflectance mode. Raman spectral profiles were recorded using a UV-Visible-NIR Raman spectrometer (Horiba Jobin Yvon LabRAM HR-800) with 514 nm laser line, 1800 grating monochromator, and a ×100 objective. For the surface analysis before and after electrochemistry, X-ray photoelectron spectroscopy (XPS) data were obtained using an XPS spectrometer (Thermo-VG Scientific K-Alpha) equipped with a hemispherical energy analyzer and a monochromated Al *K*α X-ray (1486.6 eV) source.

### 2.3 Photoelectrochemical CO_2_ Reduction and Product Analysis

Electrochemical experiments were performed in a three-electrode system using a WPG100 potentiostat/galvanostat (WonATech Co., Ltd.) instrument. The size of a working electrode was 30 mm × 5 mm, and a Pt coil (1 mm thick) and an Ag/AgCl (3.0 M KCl) were used as counter and reference electrodes, respectively. An airtight glass cell size was 100 and 50 ml of 0.1 M NaHCO_3_ (or 0.1 M KHCO_3_) electrolyte was used. Before the experiments, the electrolyte was fully bubbled with CO_2_ gas (99.999%) for obtaining CO_2_-saturated electrolyte. After that, amperometry was running at a fixed potential for 1 h under dark or 365 nm light (171.84 mW cm^−2^) conditions.

Gas and liquid products were examined by gas chromatography (GC) and nuclear magnetic resonance (NMR) spectroscopy, respectively. For gas products in an airtight closed cell, 0.5 ml of gas was taken and injected into a GC system (YL 6500, Young In Chromass Co., Ltd.) equipped with 40/60 Carboxen-1000 column, HP-Plot Q-PT column, an Ni catalyst methanizer assembly, a thermal conductivity detector, and a flame ionization detector. For liquid products, 0.5 ml of liquid electrolyte and an internal reference of 0.1 ml of DMSO/D_2_O (v/v = 1:20,000) solution were used in a 5-mm NMR Tube (WG-1241-7, Wilmad-Labglass), and then a 600-MHz FT-NMR (AVANCE III, Bruker Corp.) was employed with a water suppression method.

## 3 Results and Discussion

### 3.1 Crystal Phases and Morphologies of ZnO_NR_ and Sandwiched GZO/In/ZnO_NR_ Electrodes

**FIGURE 1:**
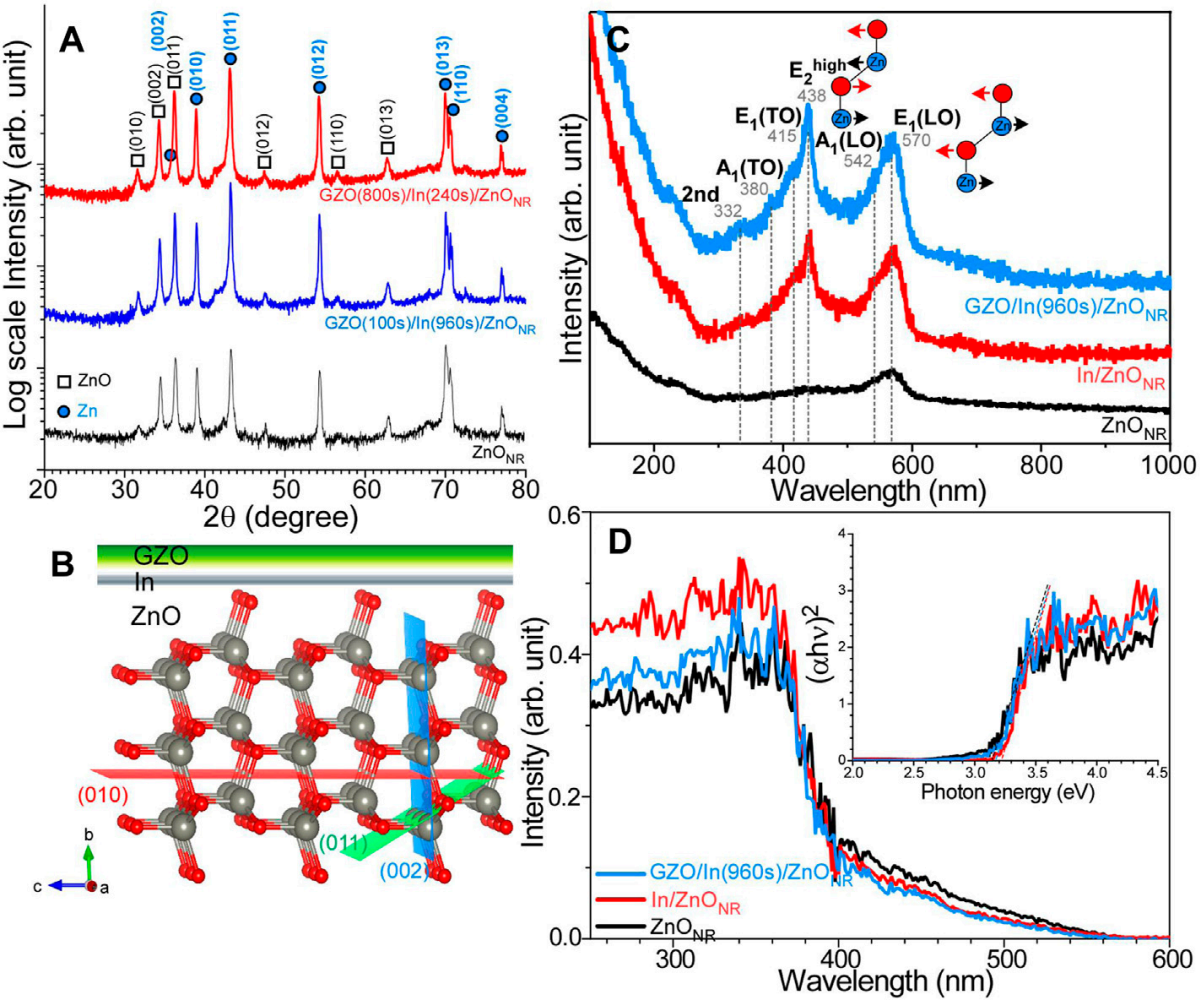
XRD profiles **(A)**, crystal phase projections **(B)**, Raman spectra **(C)**, and UV-visible absorption spectra **(D)** of bare ZnO_NR_, In/ZnO_NR_, and GZO/In/ZnO_NR_ electrode samples. Inset figure in **(D)** shows the plots of (*αhν*)^2^ vs. *hv*.

Figure 1A displays the XRD profiles for selected ZnO_NR_, GZO(100s)/In(960s)/ZnO_NR_, and GZO(800s)/In(240s)/ZnO_NR_ electrode samples. It was shown that all the XRD profiles were very similar, indicating that the XRD signals were mainly due to ZnO_NR_ support. The XRD signals were observed to be analyzed into two crystal phases of Zn and ZnO. For hexagonal phase ZnO (ref. # 98-002-9272), the corresponding peaks () were observed at 2*θ* = 31.8°, 34.5°, 36.3°, 47.7°, 56.7°, and 63.0°, assigned to the (010), (002), (011), (012), (110), and (013) crystal planes, respectively (Choi et al., 2015; Daiyan et al., 2020; Luo et al., 2020). For hexagonal metallic Zn (ref. #. 98-065-3505) (Qin et al., 2018), the corresponding peaks (blue closed circles) were observed at 2*θ* = 36.4°, 39.1°, 43.3°, 54.5°, 70.3°, 70.8°, 70.8°, and 77.3°, attributed to the crystal planes of (002), (010), (011), (012), (013), (110), and (004). No significant XRD patterns of In and GZO were observed, indicating that In and GZO were ultrathin and/or amorphous and undetectable by XRD. This is discussed further below. The crystal projection of ZnO is shown with major crystal planes in Figure 1B.

Figure 1C shows the Raman spectra of the ZnO_NR_, GZO(100s)/In(960s)/ZnO_NR_, and In(240s)/ZnO_NR_ electrode samples. All the Raman peaks were mainly assigned to those of wurtzite ZnO, where six Raman active modes are Γ = 2A1 + 2E1 + 2E2. The E1 and A1 are polar modes with transverse optical (TO) and longitudinal optical (LO) components, while the E2 modes are non-polar low and high modes. For bare ZnONR, the LO peaks were main and observed around 542 and 570 cm^−1^, assigned to A_1_(LO) and E_1_(LO) modes, respectively (Russo et al., 2014). The TO and E2 modes were observed to be weaker than those of LO components. Interestingly, upon sputter deposition of In on ZnO_NR_, the E_2_^high^ mode was substantially increased at 438 cm^−1^. The E_2_^high^ and E_1_(LO) modes are depicted in the inset of Figure 1C. The Raman profile of In(240s)/ZnO_NR_ was the same as those of the GZO(100s)/In(960s)/ZnO_NR_ sample. The peaks at 378 and 410 cm^−1^ were assigned to A_1_(TO) and E_1_(TO) modes. The other at 332 cm^−1^ was assigned to E_2_^high^−E_2_^low^ combination (Russo et al., 2014; Luo et al., 2020).

Figure 1D displays UV-visible reflectance absorption spectra (after baseline correction) of ZnO_NR_, In(240s)/ZnO_NR_, and GZO(100s)/In(960s)/ZnO_NR_ electrode samples. The absorption edge was commonly observed around 400 nm, an indication that the band gap showed no critical difference upon deposition of In and GZO. On the basis of inset plots of (*αhν*)^2^ vs. h*ν* (photon energy in eV), the band gap of bare ZnO_NR_ was estimated to be 3.14 eV and slightly increased to 3.22 and 3.17 eV for In(240s)/ZnO_NR_, and GZO(100s)/In(960s)/ZnO_NR_ electrode samples, respectively. Upon further increasing of GZO the band gap was not critically changed. This indicates that the band gap was mainly due to ZnO_NR_ and the overlayers of In and GZO showed very minor effect (Supplementary Figure S1). This is in good consistency with the XRD and Raman results.

### 3.2 Morphologies and Compositions

**FIGURE 2:**
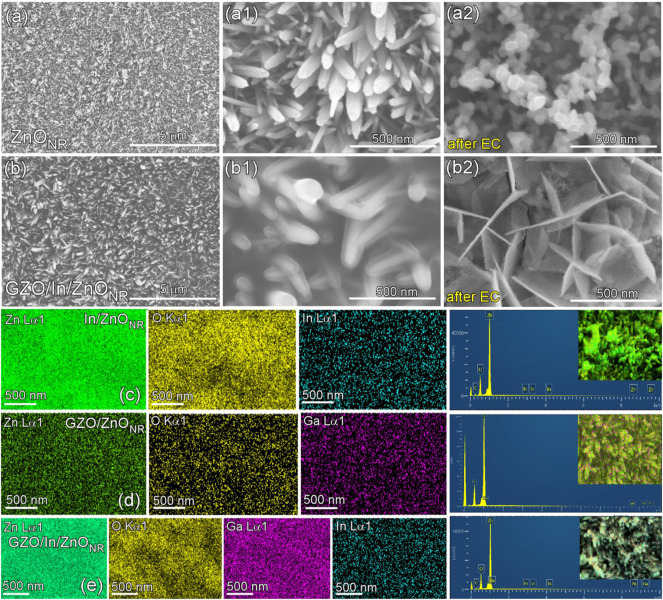
SEM images of bare ZnO_NR_ **(A, A1, A2)** and GZO(100s)/In(960s)/ZnO_NR_ **(B, B1, B2)** electrodes before and after electrochemical (EC) tests. EDX elemental mapping images and the corresponding spectra for In(240s)/ZnO_NR_ **(C)**, GZO(100s)/ZnO_NR_ **(D)**, and GZO(800s)/In(240s)/ZnO_NR_ **(E)** samples.

SEM images of ZnO_NR_ and GZO(100s)/In(960s)/ZnO_NR_ are shown in Figure 2. For bare ZnO_NR_, rods (with thickness of 10–50 nm) were observed to be vertically grown on the Zn plate (Figures 2A,A1). Upon deposition of In and GZO, the rods appeared to be fully coated by the sputter-deposited materials (Figures 2B,B1). It was commonly shown that the morphology appeared to be changed after electrochemical experiments as shown in Figures 2A2,B2. This morphology change was plausibly due to reduction of ZnO to metallic Zn during the CO_2_ reduction at a high negative potential (Luo et al., 2020). Luo et al. (2020) observed the same and explained this behavior to be due to ‘dissolution-reduction-crystallization process’ where ZnO was changed initially to soluble Zn(OH)_4_^2−^ ions followed by reduction to metallic Zn. The soluble product was observed during linear sweep voltammetry tests (Supplementary Figure S2). Surface composition elements of the prepared electrodes were examined by EDXS; Zn (0.884, 0.906, 1.012, 8.639, and 9.572 keV), O (0.525 keV), C (0.277 keV), Ga (0.957, 0.984, 1.098, and 9.252 keV), and In (0.366, 2.904, 3.113, 3.287, 3.487, and 4.161 keV) (National Institute of Standards and Technology, 2021). Elemental mapping images are shown in Figures 2C–E for In(240s)/ZnO_NR_, GZO(100s)/ZnO_NR_, and GZO(800s)/In(240s)/ZnO_NR_, respectively. Zn and O were main elements, and In (e.g., 0.04 at% for GZO(800s)/In(240s)/ZnO_NR_) and Ga (e.g., 0.66 at% for GZO(800s)/In(240s)/ZnO_NR_) were very weakly detected in the EDXS profiles. The overlayer elements were further confirmed by XPS below.

### 3.3 Electrochemical CO_2_ Reduction Tests

Electrochemical CO_2_ reduction experiments were performed at various conditions of GZO thickness, In spacer thickness, applied potentials, electrolytes, concentrations, and light irradiation.

#### 3.3.1 GZO Thickness Effects

**FIGURE 3:**
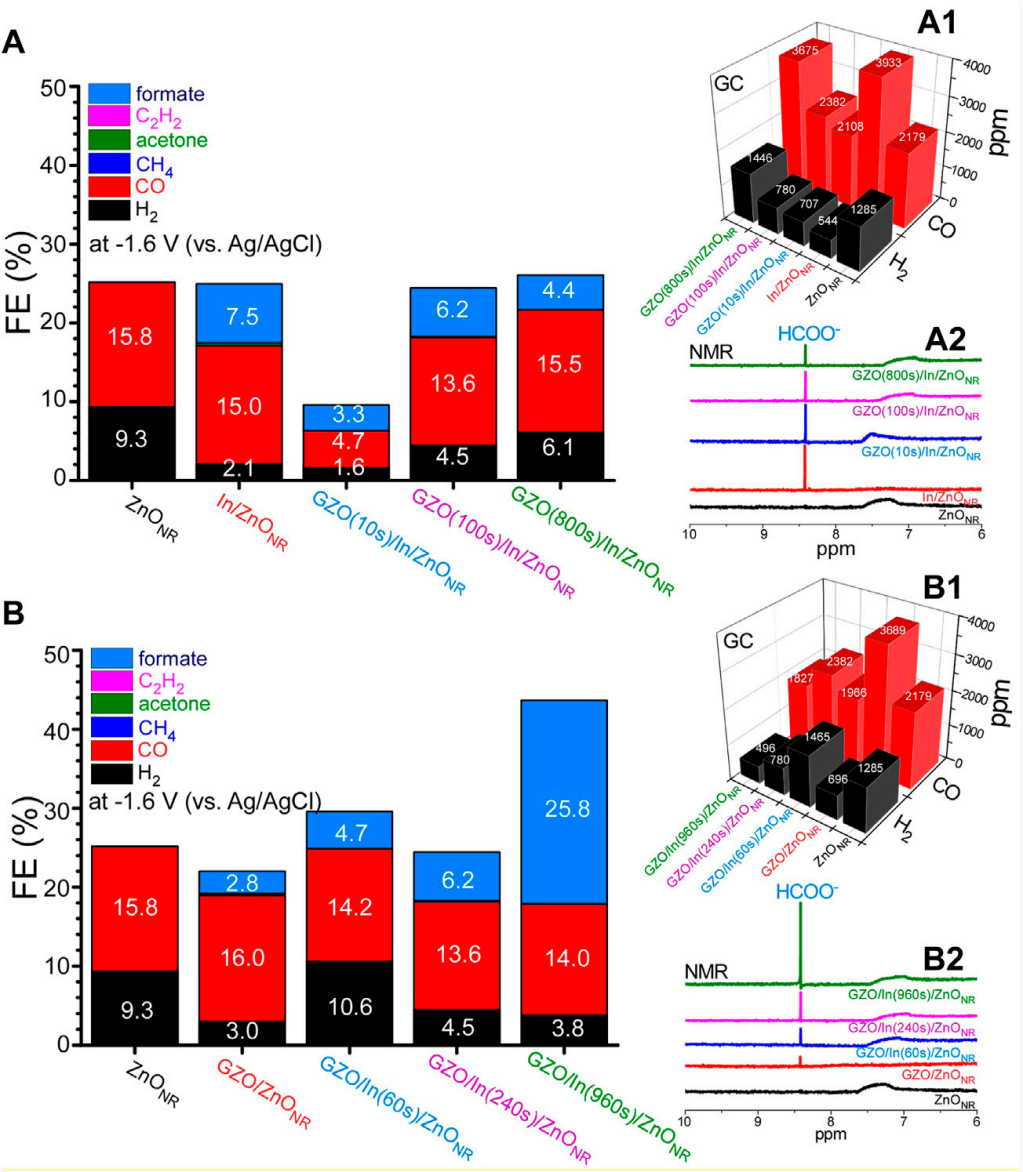
Electrochemical CO_2_ reduction FE(%) **(A)**, ppm amounts of H_2_ and CO **(A1)**, and the corresponding NMR data **(A2)** for ZnO_NR_, In(240s)/ZnO_NR_, and GZO/In(240s)/ZnO_NR_ with GZO thicknesses of 10, 100, and 800 s at −1.6 V (vs. Ag/AgCl). FE (%) **(B)**, ppm amounts of H_2_ and CO **(B1)**, and the corresponding NMR data **(B2)** for ZnO_NR_, GZO(100s)/ZnO_NR_, GZO(100s)/In/ZnO_NR_ with In thicknesses of 60, 240, and 960 s at −1.6 V (vs. Ag/AgCl).

In this experiment, the In spacer thickness was fixed and the thickness of the topmost GZO layer was changed. Figures 3A,A1,A2 display FE(%), ppm amounts of H_2_ and CO, and the corresponding NMR data, respectively, obtained for ZnO_NR_, In(240s)/ZnO_NR_, and GZO/In(240s)/ZnO_NR_ with GZO thicknesses of 10, 100, and 800 s at −1.6 V (vs. Ag/AgCl) in a 0.1 M NaHCO_3_ electrolyte for 1 h. For bare ZnO_NR_, major gaseous products were observed to be H_2_ (1285 ppm) and CO (2179 ppm) with Faradaic efficiencies (FEs) of 9.3 and 15.8%, respectively, and a CO/H_2_ ratio of 1.7. One minor species included CH_4_ with 1.5 ppm. The CH_4_ amount was not significantly dependent on the GZO thickness (Supplementary Table S1). No liquid products were observed. Total FE (%) was observed to be below 30%. Several reasons were plausibly proposed. In the present study, we used a 2 mm thick Zn plate different from those (e.g., powder in carbon paste) in the literature (Sekimoto et al., 2016; Tatsumi et al., 2017; Kikkawa et al., 2018; Lu et al., 2018; Qin et al., 2018; Yamamoto et al., 2018; Zhang et al., 2018; Liu et al., 2019; Ma et al., 2019; Murali et al., 2019; Zhang et al., 2019; Akatsuka et al., 2020; Daiyan et al., 2020; Hou et al., 2020; Luo et al., 2020; Tan et al., 2020; Yang et al., 2020; Deng et al., 2021; Li et al., 2021; Liang et al., 2021; Ma et al., 2021; Teng et al., 2021; Wang et al., 2021; Wei et al., 2021; Wu et al., 2021; Xiao et al., 2021; Yoon et al., 2021). Therefore, non-Faradaic current such as capacitive current might be significantly involved. As discussed previously in Figure 2, ZnO reduction occurred, and therefore ZnO reduction current was also added in the amperometry current. Further, the experiment was performed in a single cell and therefore evolved oxygen gas was present in the cell. Therefore, oxygen reduction reaction current was also plausibly involved (Zhou et al., 2020).

Upon deposition of In, drastic changes were observed; H_2_ production was substantially decreased while formate was newly observed with a substantial amount. The NMR data (Figure 3A2) confirmed the formation of formate. The FEs (%) for H_2_, CO, and formate over In(240s)/ZnO_NR_ were estimated to be 2.1, 15.0, and 7.5%, respectively. The CO/H_2_ ratio was substantially increased and estimated to be 7.2. Moreover, C_2_H_2_ was very meaningfully observed with an amount of 16.7 ppm (Supplementary Table S1). Upon deposition of a small amount of GZO on In(240s)/ZnO_NR_ for 10 s, the FEs(%) of H_2_, CO, and formate were observed to be decreased. After GZO deposition for 100 s, the FEs(%) of H_2_, CO, and formate were all increased. Upon further increasing GZO for GZO(800s)/In(240s)/ZnO_NR_, the FEs(%) of H_2_ and CO were increased by ×1.36 and ×1.14. On the other hand, the formate was decreased by ×0.71. The CO/H_2_ ratio was estimated to be 2.5. Conclusively, In was a key factor of producing formate and the GZO thickness was a control factor for controlling the amounts of three major products (H_2_, CO, and formate).

#### 3.3.2 In Spacer Thickness Effects

For the electrodes in this experiment, the topmost GZO layer was fixed and the In spacer thickness was changed. Figures 3A,A1,A2 show FE(%), ppm amounts of H_2_ and CO, and the corresponding NMR data for ZnO_NR_, GZO(100s)/ZnO_NR_, and GZO(100s)/In/ZnO_NR_ with In thicknesses of 60, 240, and 960 s at −1.6 V (vs. Ag/AgCl) in 0.1 M NaHCO_3_ electrolyte for 1 h. Upon deposition of GZO on ZnO_NR_ for 100 s, formate was newly detected with an FE(%) of 2.8%. The formate enhancement was smaller than that (7.5%) after In deposition. This also confirms that In was more efficient for formate production, in good consistency with the literature (Ma et al., 2019; Murali et al., 2019; Zhang et al., 2019; Hou et al., 2020; Yang et al., 2020; Li et al., 2021; Ma et al., 2021; Teng et al., 2021; Wei et al., 2021; Wu et al., 2021; Xiao et al., 2021). The FE(%) of CO was slightly increased while that of H_2_ was substantially decreased by ×0.32, compared with those for bare ZnO_NR_. Therefore, the CO/H_2_ ratio was increased to 5.3 (Supplementary Table S2). One solid interesting observation was that the formate was continuously increased as the thickness of In spacer was increased, confirmed by NMR data in Figure 2B2. For CO production, when In was sandwiched, the FE(%) was observed to be somewhat decreased and lower than those of ZnO_NR_ and GZO(100s)/ZnO_NR_. For H_2_ production, the FE(%) of H_2_ was maximum when In spacer thickness was 10 s, but the FEs(%) for other samples was observed to be lower than that of bare ZnO_NR_. The CH_4_ amount (with only 1.3–1.9 ppm) was also not significantly dependent on the In spacer thickness (Supplementary Table S2). C_2_H_2_ was meaningfully detected with amounts of 2.2–4.1 ppm when In thickness was above 240 s (Supplementary Table S2). Acetone was very weakly detected. Reliability and repeatability were examined by showing errors for H_2_ and CO products in the repetitive experiments (Supplementary Table S3) and showed no significant impact on the conclusion.

#### 3.3.3 Applied Potential Effects

**FIGURE 4:**
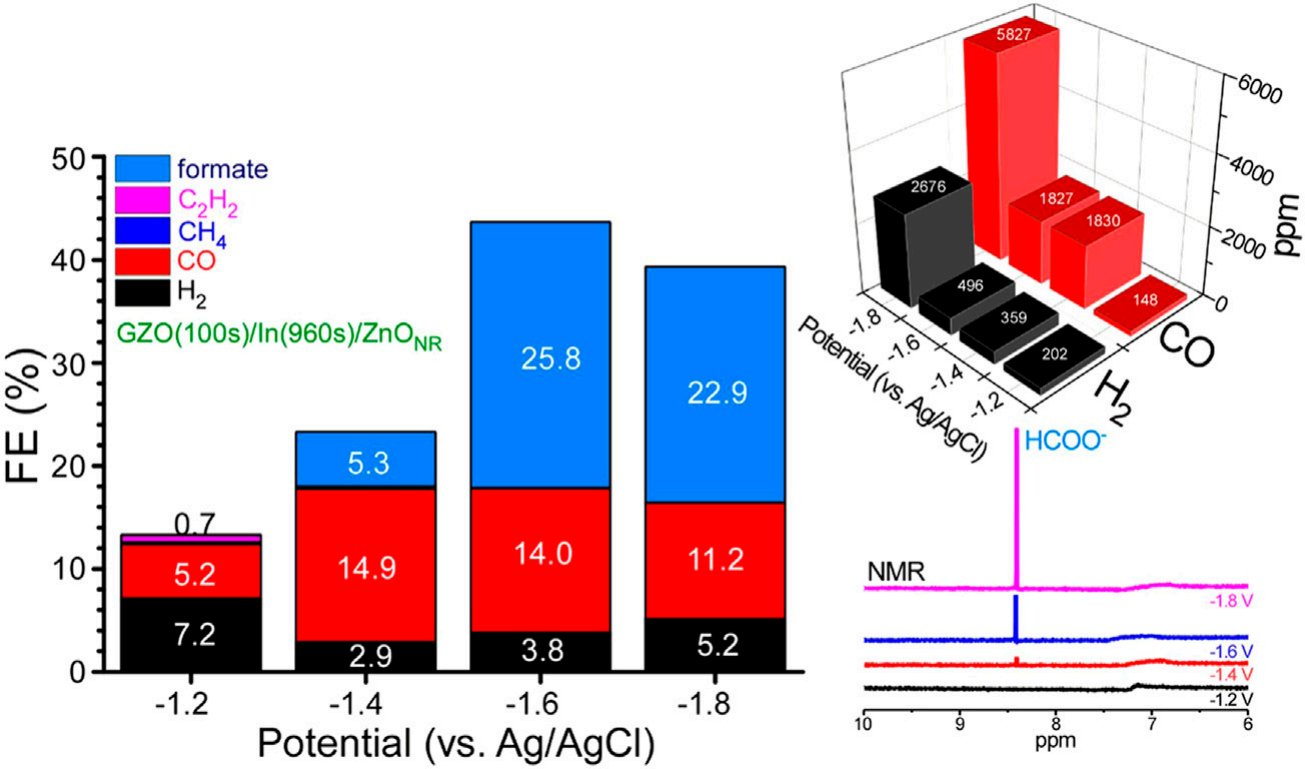
Electrochemical CO_2_ reduction FE(%), ppm amounts of H_2_ and CO, and the corresponding NMR data for GZO(100s)/In(960s)/ZnO_NR_ at different applied potentials of −1.2, −1.4, −1.6, and −1.8 V (vs. Ag/AgCl).

A GZO(100s)/In(960s)/ZnO_NR_ electrode sample was selected and tested at different applied potentials of −1.2, −1.4, −1.6, and −1.8 V (vs. Ag/AgCl) in 0.1 M NaHCO_3_ electrolyte for 1 h (Figure 4; Supplementary Table S4). At a low potential of −1.2 V, gaseous products (H_2_, CO, and C_2_H_2_) were mainly produced and no formate was observed as seen in the NMR spectrum. The CH_4_ amount (with only 1.3–1.8 ppm) was not significantly changed with applied potential (Supplementary Table S4). The CO/H_2_ ratio was estimated to be 0.7 at −1.2 V. Upon increasing the potential to −1.4 V formate was clearly increased with an FE(%) of 5.3%. The amount of formate was continuously increased with increasing the potential as seen in the NMR data. Upon further increasing the potential to −1.6 and −1.8 V FEs(%) were observed to be 25.8 and 22.9%, respectively. CO and H_2_ productions were also increased with increasing the potential and the amounts (ppm) were highest at −1.8 V. After considering the current density at each applied potential the FE(%) of CO at −1.4 V was obtained to be 14.9 and higher. The FE(%) became decreased with increasing the potential. The FE(%) of H_2_ at −1.4 V was minimum of 2.9 but increased with increasing the potential. In other words, when the FE(%) of CO was decreased the FE(%) of H_2_ was inversely increased.

#### 3.3.4 Photoirradiation Effects

**FIGURE 5:**
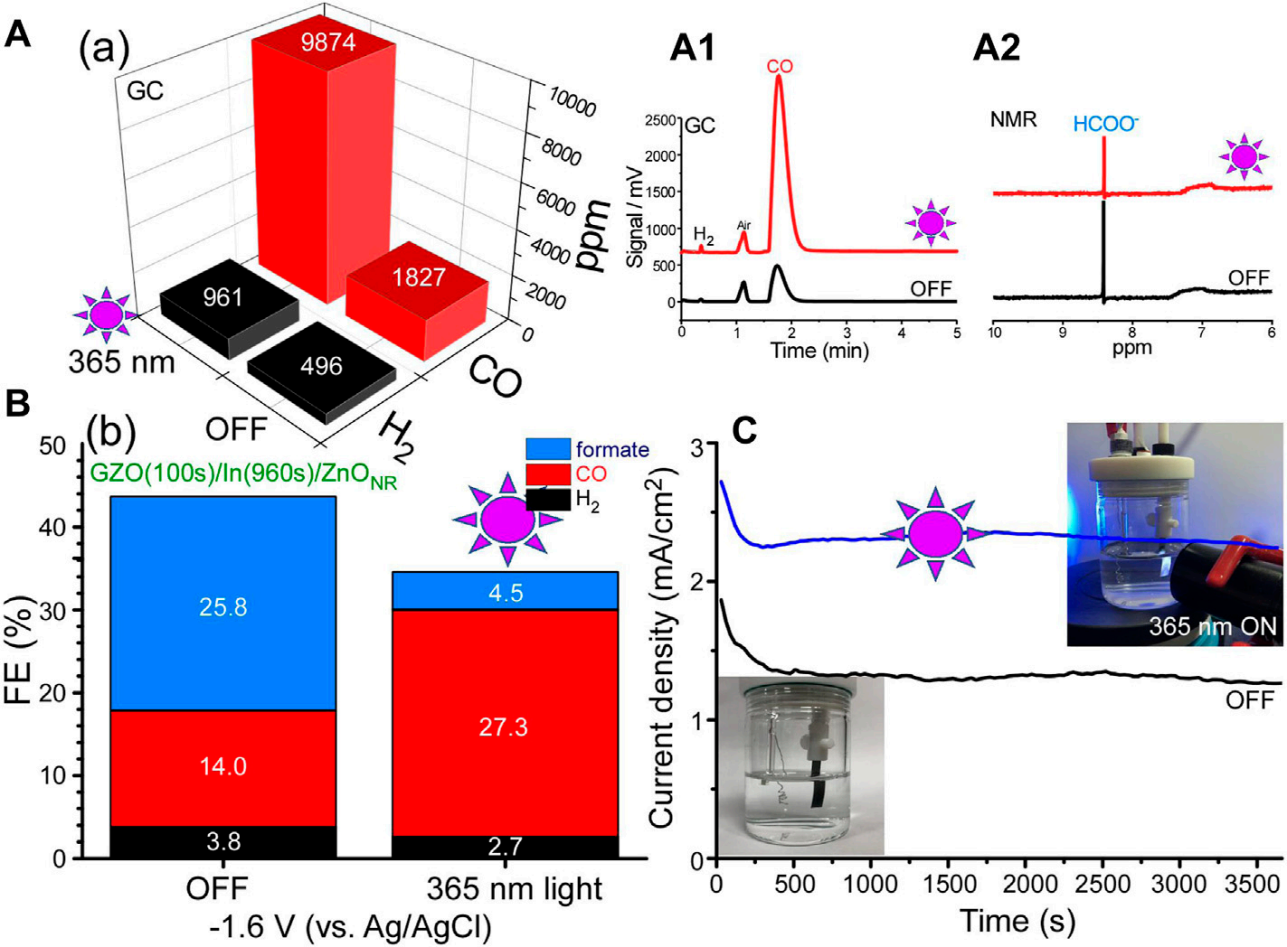
Photoelectrochemical CO_2_ reduction ppm amounts **(A)** of H_2_ and CO, GC **(A1)** and NMR **(A2)** data, the corresponding FE(%) **(B)**, and amperometry *i-t* profiles for GZO(100s)/In(960s)/ZnO_NR_ electrode under dark and 365 nm light conditions **(C)**. Inset photos show the electrochemical cells under dark and 365 nm light.

Light (365 nm) ON and OFF conditions were tested to examine photoirradiation effects on photoelectrochemical CO_2_ reduction over a selected GZO(100s)/In(960s)/ZnO_NR_ electrode (Figure 5). The band gap edge was observed around 400 nm and therefore a shorter wavelength of 365 nm was chosen in this experiment. The amounts (ppm) of H_2_ and CO were commonly increased upon irradiation of 365 nm light during amperometry (Figure 5A). The amount of H_2_ was increased by ×1.93 and that of CO was increased by ×5.40 (Figures 5A,A1). However, as seen in the NMR spectra (Figure 5A2), the formate was decreased after light irradiation. The FE(%) of formate was drastically decreased by ×0.17 (Figure 5B). Based on the amperometry *i-t* curve (Figure 5C), the current density was increased by about ×1.7 upon irradiation of 365 nm light. In the FE(%) calculation, the total current (including photogenerated current) was used. The FE(%) of H_2_ was decreased from 3.8 to 2.7% after light irradiation. The FE(%) of CO was increased from 14.0 to 27.3% after light irradiation, increased by a factor of ×1.95. Consequently, CO/H_2_ ratio was increased from 3.7 to 10.3. C_2_H_2_ was newly detected with an amount of 4.8 ppm. Further, CH_4_ was increased from 1.3 to 4.9 ppm (Supplementary Table S5).

A longer wavelength of 405 nm light was also used, and the wavelength was near the band gap edge (Supplementary Figure S3). For GZO(100s)/In(960s)/ZnO_NR_ electrode, the FE(%) of CO was increased by a factor of ×1.47 under 405 nm irradiation. This was less compared with ×1.95 for 365 nm light. This indicates that the 405 nm light showed less impact on producing CO. Inversely, similar phenomena were observed for formate and H_2_. The FE(%) of formate was decreased by ×0.81 and that of H_2_ was decreased by ×0.79 under 405 nm light. These diminutions were less compared with those (×0.17 for formate and ×0.79 for H_2_) under 365 nm light. It was a common observation that CO production was enhanced under light, but formate production was negated by light irradiation. When light wavelength was shorter, this observation was more pronounced. For GZO(100s)/In(240s)/ZnO_NR_ electrode (Supplementary Figure S3), similarly CO production was increased and formate production was decreased under light. However, unlike the GZO(100s)/In(960s)/ZnO_NR_ electrode, the FE(%) of H_2_ was also increased under light. This was due to a smaller amount of In spacer and the H_2_ production was more or less affected by the overlayer GZO. For GZO(10s)/In(240s)/ZnO_NR_ electrode, the thickness of GZO was much less. Consequently, the production of H_2_ was observed to be much less than those for other samples with GZO thickness for 100 s. Similarly, CO production was also increased under light. Conclusively, it was evident that CO was increased under light, and formate and H_2_ were determined by the thicknesses of GZO and the In spacer.

#### 3.3.5 Electrolyte and Electrolyte Concentration Effects

Electrolytes of 0.1 M KHCO_3_ and a higher concentration of 0.5 M NaHCO_3_ were also tested for a selected GZO(100s)/In(960s)/ZnO_NR_ electrode and provided in Supplementary Figure S4. When the electrolyte was changed from 0.1 M NaHCO_3_ to 0.1 M KHCO_3_ it was observed that the amounts (ppm) of H_2_ and CO were increased from 496 to 804 ppm and from 1827 to 4,422 ppm, respectively. However, because of a current increase in 0.1 M KHCO_3_ the FEs(%) of formate, CO and H_2_ were decreased by ×0.81, ×0.91, and ×0.61, respectively. C_2_H_2_ and acetone were newly but very weakly detected (Supplementary Table S6). When the electrolyte concentration was increasing from 0.1 to 0.5 M NaHCO_3_ the H_2_ production was dramatically increased from 496 to 5,563 ppm by a factor of ×11.2. The CO production was increased from 1,827 to 4,695 ppm by a factor of ×2.57. When the current density was considered, the FEs(%) of formate and CO were decreased by ×0.24 and ×0.51, respectively. On the other hand, the FE(%) of H_2_ was increased from 3.8 to 8.4% by a factor of 2.2. Consequently, the CO/H_2_ ratio was observed to be 0.8. Further, C_2_H_2_ was detected with an amount of 12 ppm.

Although similar sandwiched samples have not been reported, some literature was summarized and discussed in Supplementary Table S7. Although the FE(%) was somewhat less, compared with those in the literature, the present study showed very unique information on sandwiched structures under various experimental conditions.

### 3.4 X-Ray Photoelectron Spectroscopy Before and After CO_2_ Reduction Tests

#### 3.4.1 XPS With GZO Thickness

**FIGURE 6:**
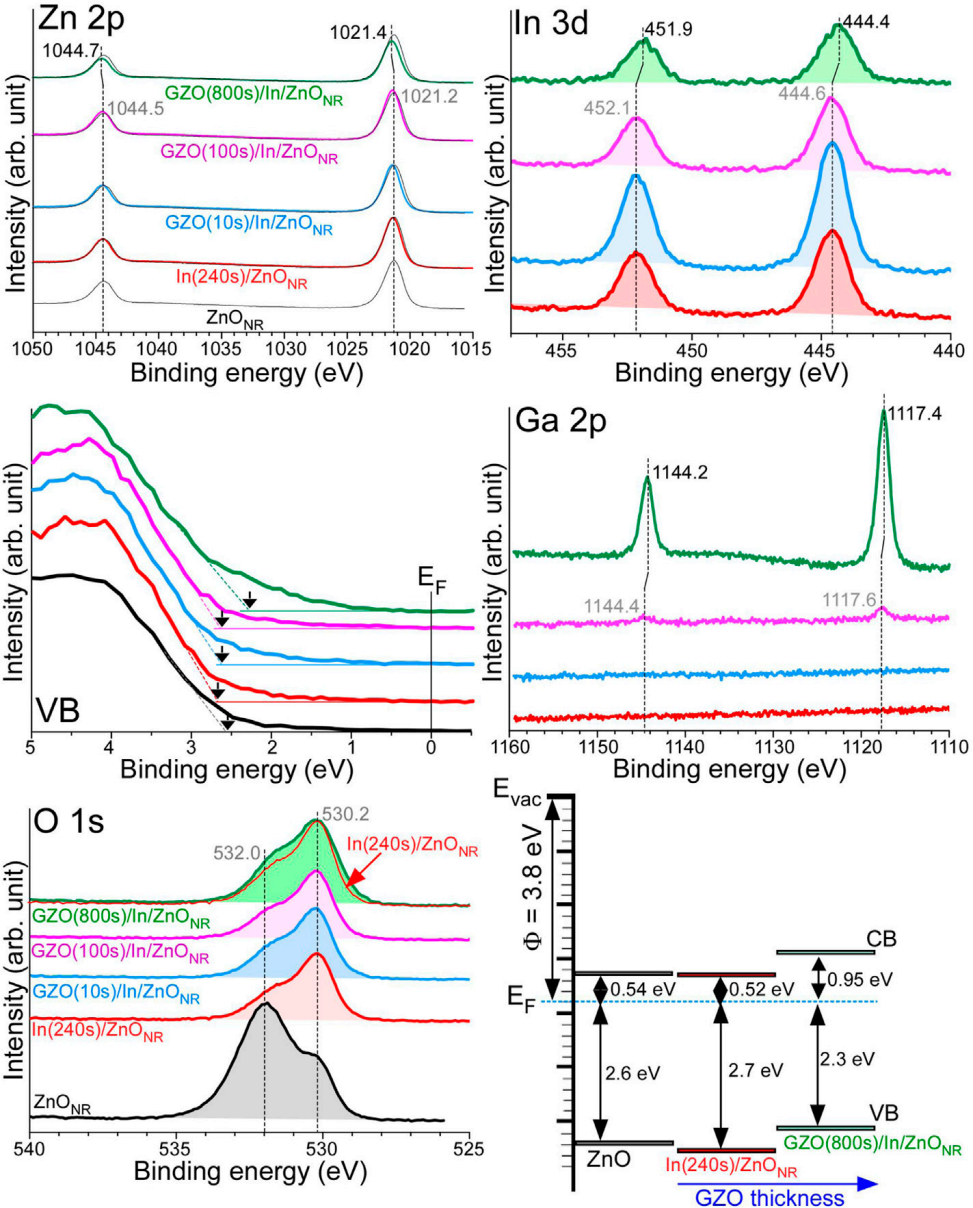
Zn 2p, In 3d, VB, Ga 2p, and O 1s XPS profiles for ZnO_NR_, In(240s)/ZnO_NR_, GZO/In(240s)/ZnO_NR_ with GZO thicknesses of 10, 100, and 800 s. The interfacial energy level alignments for ZnO_NR_, In(240s)/ZnO_NR_, and GZO(800s)/In(240s)/ZnO_NR_ samples.

Figure 6 displays Zn 2p, In 3d, valence band (VB), Ga 2p, O 1s XPS profiles, and the consequent interfacial energy levels for ZnO_NR_, In(240s)/ZnO_NR_, GZO/In(240s)/ZnO_NR_ with GZO thicknesses of 10, 100, and 800 s. For bare ZnO_NR_, Zn 2p_3/2_ and Zn 2p_1/2_ peaks were observed at 1021.2 and 1044.5 eV, respectively, with a spin-orbit (S-O) splitting of 23.3 eV. Upon deposition of In, the Zn 2p binding energy (BE) positions showed no critical change and the width of Zn 2p peak became slightly narrower. As the GZO was deposited and the thickness was increased Zn 2p BE became shifted to a higher BE position. For GZO(800s)/In(240s)/ZnO_NR_ electrode, Zn 2p_3/2_ and Zn 2p_1/2_ peaks were shifted by +0.2 eV and observed at 1021.4 and 1044.7 eV, respectively (Naumkin et al., 2012; Ansari et al., 2013; Choi et al., 2015; Kang et al., 2019). For In(240s)/ZnO_NR_ electrode, the In 3d_5/2_ and In 3d_3/2_ XPS profiles were observed at 444.6 and 452.1 eV, respectively with an S-O splitting energy of 7.5 eV (Naumkin et al., 2012; Li et al., 2021). The BE positions were shifted to lower BEs by −0.2 eV, and observed at 444.4 and 451.9 eV, respectively, for GZO(800s)/In(240s)/ZnO_NR_ electrode. For the Ga 2p XPS profiles at low coverage, Ga 2p_3/2_ and Ga 2p_1/2_ peaks were observed at 1117.6 and 1144.4 eV, respectively, with a spin-orbit S-O splitting of 26.8 eV (Naumkin et al., 2012; Akatsuka et al., 2020; Yoon et al., 2021). The BEs were shifted to lower BE positions by −0.2 eV, and observed at 1117.4 and 1144.2 eV, respectively, for GZO(800s)/In(240s)/ZnO_NR_ electrode. Conclusively, Zn 2p XPS peaks were shifted to higher BE positions while In 3d and Ga 2p BEs were shifted to lower BE positions. GZO (Ga_2_O_3_ and ZnO) was sputter-deposited on top of In(240s)/ZnO_NR_ for a very long time of 800 s. During this time, Ga, Zn, and In reacted, and consequently Zn 2p was shifted to a higher BE position while In 3d and Ga 2p were shifted to lower BE positions.

For the corresponding O 1s XPS profiles, two broad O 1s peaks were commonly observed at 530.2 and 532.0 eV that were attributed to lattice and surface oxygens, respectively. The surface oxygen peak at 532.0 eV was observed to be stronger for bare ZnO_NR_ while the lattice O 1s peak at 530.2 eV was stronger for the other samples. The O 1s peaks for GZO(800s)/In(240s)/ZnO_NR_ electrode were broader than those for In(240s)/ZnO_NR_ electrode. The broader O 1s signals were plausibly due to the topmost GZO layer. In other words, more different oxygen species were intuitively present in the GZO(800s)/In(240s)/ZnO_NR_ electrode, compared with the In(240s)/ZnO_NR_ electrode.

In the corresponding VB spectra of ZnO_NR_, the VB edge appeared at 2.6 eV below the Fermi level (E_F_). Upon deposition of In, the VB edge was observed around 2.7 eV below the E_F_. The conduction band (CB) edge was similar based on the corresponding UV-Vis absorption spectra in Figure 1 and the VB spectra. By increasing the coverage of GZO, it appeared that the VB edge was shifted toward the Fermi level. For the GZO(800s)/In(240s)/ZnO_NR_ electrode, the VB edge was observed at 2.3 eV below the E_F_ and the CB was observed at 0.95 eV above the E_F_. For the energy band diagram, we assumed the multilayer system to be a single well-hybridized system.

The overlayer thickness was roughly estimated using a well-known equation (Powell and Jablonski, 2000; Naumkin et al., 2012), *I* = *I*_0_exp(-d/λ), where *I* and *I*_*o*_ is the Zn 2p XPS intensities before and after overlayer deposition, d is the overlayer thickness, and *λ* is the electron inelastic mean free path (here *λ* = 1.5 nm used) (Naumkin et al., 2012), respectively. The overlayer thicknesses were calculated to be approximately 0.1 nm for In deposition of 240 s and approximately 0.4 nm for GZO deposition of 800 s, respectively (Powell and Jablonski, 2000).

#### 3.4.2 XPS With In Spacer Thickness

**FIGURE 7:**
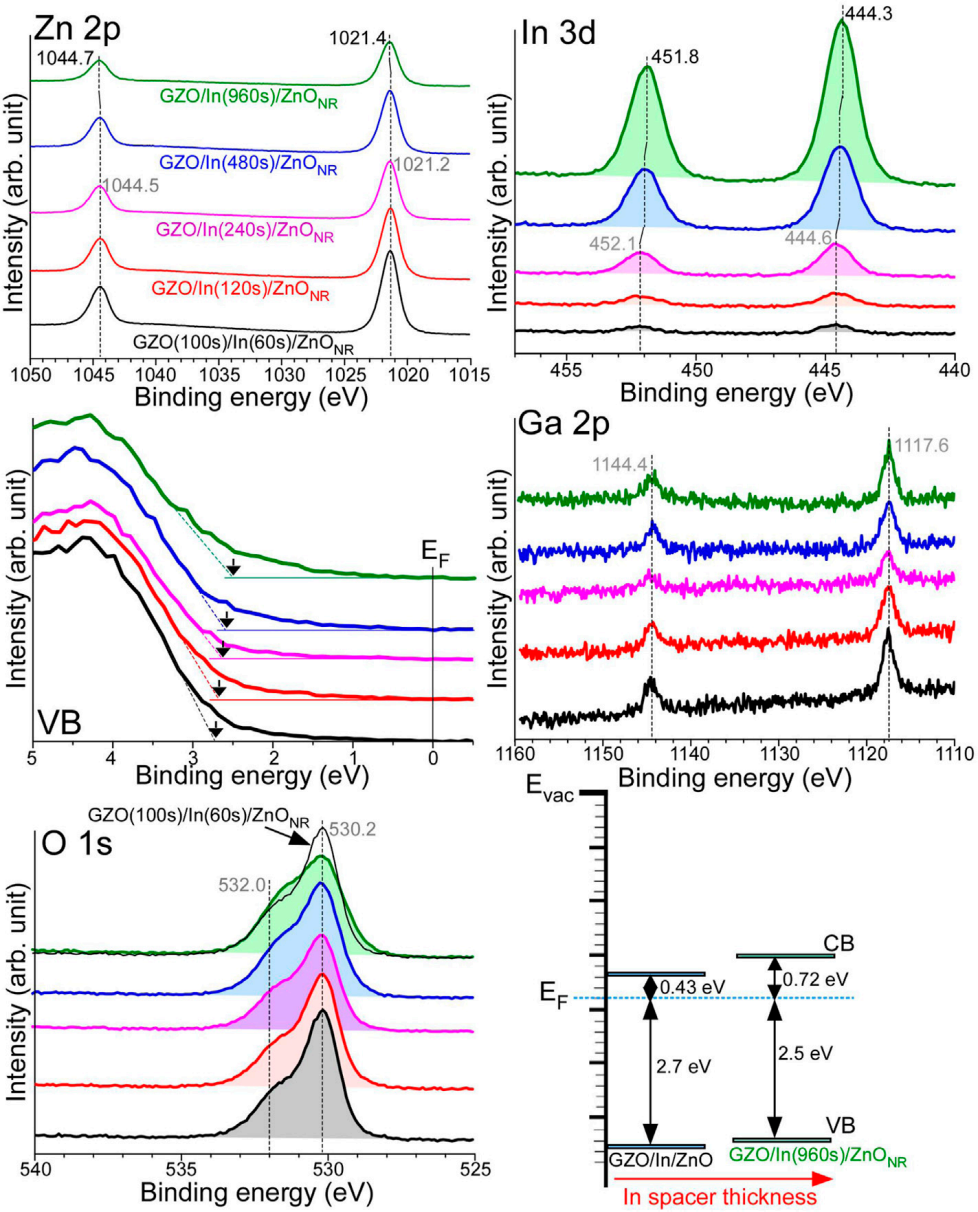
Zn 2p, In 3d, VB, Ga 2p, and O 1s XPS profiles for GZO(100s)/In/ZnO_NR_ with In thicknesses of 60, 120, 240, 480, and 960 s. The interfacial energy level alignments for GZO(100s)/In(60s)/ZnO_NR_ and GZO(100s)/In(960s)/ZnO_NR_ samples.

Figure 7 displays Zn 2p, In 3d, VB, Ga 2p, O 1s XPS profiles, and the interfacial energy levels for GZO(100s)/In/ZnO_NR_ with In thicknesses of 60, 120, 240, 480, and 960 s. For GZO(100s)/In (60s)/ZnO_NR_, Zn 2p_3/2_ and Zn 2p_1/2_ peaks were observed at 1021.2 and 1044.5 eV, respectively, with an S-O splitting of 23.3 eV. The BE positions were the same as those for bare ZnO_NR_. As the sandwiched In thickness was increased Zn 2p BE became shifted to a higher BE position. For GZO(100s)/In(960s)/ZnO_NR_ electrode, Zn 2p_3/2_ and Zn 2p_1/2_ peaks were shifted by +0.2 eV and observed at 1021.4 and 1044.7 eV, respectively (Naumkin et al., 2012; Ansari et al., 2013; Choi et al., 2015; Kang et al., 2019). For GZO(100s)/In (60s)/ZnO_NR_, the In 3d_5/2_ and In 3d_3/2_ XPS profiles were observed at 444.6 and 452.1 eV, respectively, with an S-O splitting energy of 7.5 eV (Naumkin et al., 2012; Li et al., 2021). As the In thickness was increased, the In 2p intensity was increased as expected. The BE positions were shifted to lower BEs by −0.3 eV, and observed at 444.3 and 451.8 eV, respectively, for GZO(100s)/In(960s)/ZnO_NR_ electrode. It appeared that In reacted with ZnO_NR_ support during the very long time of 960 s. Therefore, Zn 2p was shifted to a higher BE position while In 3d was shifted to a lower BE position. The Ga 2p_3/2_ and Ga 2p_1/2_ peaks were commonly observed at 1,117.6 and 1,144.4 eV, respectively, with a spin-orbit S-O splitting of 26.8 eV. The BEs showed no change with the In spacer thickness. It was expected that the topmost GZO layer thickness was the same for all the samples. This indicates that GZO was not critically reacted with In during the relatively shorter deposition time of 100 s.

Two broad O 1s peaks were commonly observed at 530.2 and 532.0 eV that were attributed to lattice and surface oxygens, respectively, as discussed above. As the In thickness was increased, the lattice O 1s peak was decreased while the surface oxygen peak was observed to be somewhat enhanced. On the basis of the lattice O 1s peak and Zn 2p XPS, the XPS signals from the ZnO_NR_ support were decreased as the thickness of the In spacer was increased as expected. For the corresponding VB spectra of GZO(100s)/In (60s)/ZnO_NR_, the VB edge was observed at 2.7 eV below the E_F_. Upon increasing the thickness of the In spacer, the VB edge became slightly shifted toward the E_F_ and observed around 2.5 eV below the E_F_ for the GZO(100s)/In(960s)/ZnO_NR_ electrode. Based on a similar band gap, the interfacial energy level was depicted as shown in Figure 7.

**FIGURE 8:**
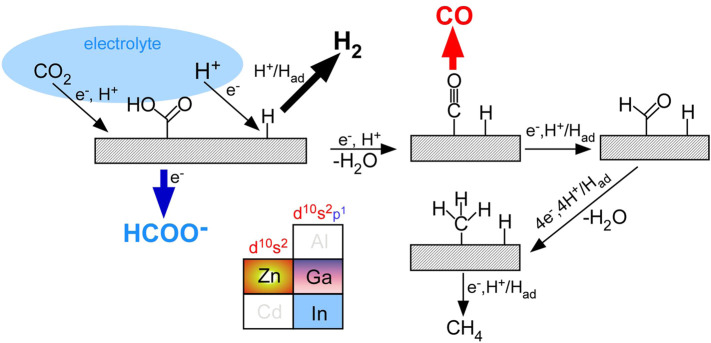
CO_2_ reduction mechanism for major CO_2_ reduction products.

On the basis of the CO_2_ reduction of major products, the simplified CO_2_ reduction mechanism is depicted in Figure 8 (Sohn et al., 2017; Wang et al., 2019; Song et al., 2020; Prabhu et al., 2020; Sun et al., 2021; da Silva Freitas et al., 2021; Ješić et al., 2021; Ochedi et al., 2021; Chen et al., 2021; Bellardita et al., 2021). In an NaHCO_3_ electrolyte saturated with CO_2_, two processes are initially involved; H^+^ + e^−^ → H_ad_ and CO_2_ + H^+^ + e^−^ → HOOC_ad_. The surface H_ad_ is liberated as gaseous H_2_ via H_ad_ + H^+^ + e^−^ → H_2_ or H_ad_ + H_ad_ → H_2_ (Sohn et al., 2017; Wang et al., 2019; Prabhu et al., 2020; Song et al., 2020; Bellardita et al., 2021; Chen et al., 2021; da Silva Freitas et al., 2021; Ješić et al., 2021; Ochedi et al., 2021; Sun et al., 2021). This process became pronounced when the thickness of GZO was increased. The surface HOOC_ad_ is then transformed into formate or changed into surface O≡C_ad_ via HOOC_ad_ + H^+^ + e^−^ → O≡C_ad_ + H_2_O (Lu et al., 2018; Qin et al., 2018; Zhang et al., 2018; Liu et al., 2019; Daiyan et al., 2020; Luo et al., 2020; Wang et al., 2021). The formate production was only observed when In and GZO (Ga_2_O_3_:ZnO) were present. Without In or GZO, no formate was produced. The formate production was more significantly dependent on In thickness. Ga and In have an electron configuration *d* ^*10*^ *s* ^*2*^ *p* ^*1*^, and it appeared that the partially filled *p* orbital may play a role in the formate production (Li et al., 2021). The surface O≡C_ad_ is likely liberated as gaseous CO. In the initial stage over GZO/In/ZnO_NR_ electrode, H_2_, formate, and CO competitively occurred.

The CO production process became dominant when light was irradiated on the electrode surface. Inversely, the formate and H_2_ production processes became reduced under light irradiation. CH_4_ and C_2_H_2_ were minor products and the net reactions are written as CO_2_ + 8H^+^ + 8e^−^ → CH_4_ + 2H_2_O and 2CO_2_ + 10H^+^ + 10e^−^ → C_2_H_2_ + 4H_2_O, respectively (Sohn et al., 2017; Wang et al., 2019; Song et al., 2020; Prabhu et al., 2020; Bellardita et al., 2021; Chen et al., 2021; da Silva Freitas et al., 2021; Ješić et al., 2021; Ochedi et al., 2021; Sun et al., 2021). Some surface O≡C_ad_ species may transform into HOC_ad_ and H_x_C_ad_. Consequently, some CH_4_ and C_2_H_2_ were expected to be produced. In the photoelectrochemical CO_2_ reduction process, light was irradiated and therefore electrons (e^−^_CB_) and holes (h^+^_VB_) are generated in the conduction and valence bands, respectively (Choi et al., 2015; Yoon et al., 2021). Plausible processes under photoirradiation include 1) e^−^_CB_ + adsorbed O_2_ → •O_2_^−^, 2) OH^−^ + h^+^_VB_ → •OH, 3) •O_2_^−^ + H^+^ → •OOH, 4) •OOH + e^−^ + H^+^ → H_2_O_2_, and 5) H_2_O_2_ + e^−^ → •OH + OH^−^. Oxygen was expected to participate in the process because the electrochemical test was conducted in a single cell and therefore evolved O_2_ gas was present in the cell. The generated active species are also expected to play roles in CO_2_ reduction process. H_2_ production was negated because of the side reactions. Surface H was consumed to be less under photoirradiation, and instead surface CO was facile to be liberated. Formate production was also observed to be diminished under UV irradiation. It was plausibly due to that formate reacted with •OH to return to •CO_2_^−^ via the reaction of HCO_2_^−^ (formate) + •OH → •CO_2_^−^ + H_2_O (Talu and Diyamandoglu, 2004).

## 4 Conclusion

Hybrid sandwiched GZO/In/ZnO_NR_ was prepared with various thicknesses of overlayer GZO and In spacer. Their electrochemical CO_2_ reduction performances and products were evaluated by gas chromatography and nuclear magnetic spectroscopy. GZO and In were not clearly detected by XRD and Raman spectroscopy, but the elements were detected by EDXS and XPS. This indicates that the overlayers of GZO and In were ultrathin and/or amorphous.

For bare ZnO_NR_, major products were observed to be H_2_ and CO by electrochemical CO_2_ reduction. Formate production was observed upon introducing the In layer and the amount was increased as the In spacer thickness was increased. When the In spacer layer was fixed and the GZO thickness was varied the FEs(%) of H_2_, CO, and formate were relatively varied. For a GZO/In/ZnO_NR_ electrode, the FE(%) of CO was decreased as the applied potential was increased from −1.4 to −1.8 V (vs. Ag/AgCl) while that of H_2_ was inversely increased with potential. The FE(%) of formate showed the highest at −1.6 V (vs. Ag/AgCl). Upon 365 nm light irradiation, CO production was significantly increased while formate was dramatically diminished. CO and formate productions were decreased while H_2_ was increased at a higher concentration of 0.5 M NaHCO_3_. In 0.1 M KHCO_3_ electrolyte, the FEs(%) of all the products were decreased, compared with those in 0.1 M NaHCO_3_. Faradaic efficiencies were all highly dependent on the relative amounts of overlayer GZO and In spacer, as well as applied potential, light irradiation, and electrolyte.

Overall, the present study provides new strategic information on the development of sandwiched hybrid electrodes for energy and the environment.

## Data Availability

The original contributions presented in the study are included in the article/Supplementary Material, further inquiries can be directed to the corresponding author.
