# Supplementary material for: Photoelectrochemical CO2 Reduction Products Over Sandwiched Hybrid Ga2O3:ZnO/Indium/ZnO Nanorods
Source: Front Chem. 2022 Feb 9;10:814766. doi: 10.3389/fchem.2022.814766 (PMC8863927; doi:10.3389/fchem.2022.814766)
Supplement: Supplementary file 1 [file DataSheet1.docx]

**Supplemental Information**

**Photoelectrochemical CO_2_ reduction products over sandwiched hybrid Ga_2_O_3_:ZnO/Indium/ZnO nanorods**

Hye Ji Jang,^1^ Ju Hyun Yang,^1,2^ Min Hee Joo,^1,2^ Ju Young Maeng, ^1^ Young Jun Kim,^1^ Choong Kyun Rhee,^1^ and Youngku Sohn^1,2,*^

*^1^Department of Chemistry, Chungnam National University, Daejeon 34134, Republic of Korea*

*^2^Department of Chemical Engineering and Applied Chemistry, Chungnam National University, Daejeon 34134, Republic of Korea*

* Corresponding author e-mail: youngkusohn@cnu.ac.kr


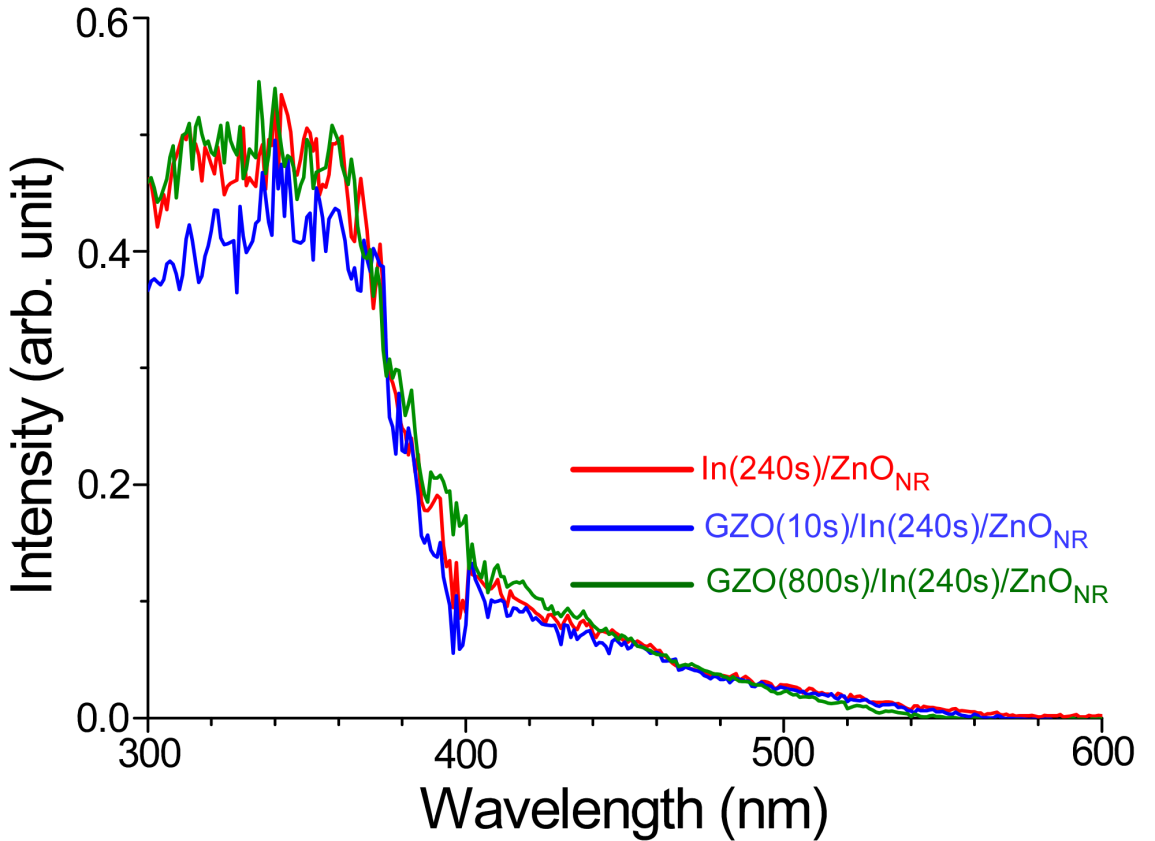


**Fig. S1**. Reflectance UV-visible absorption spectra of In(240s)/ZnO_NR_, GZO(10s)/In(240s)/ZnO_NR_, and GZO(800 s)/In(240s)/ZnO_NR_ electrode samples.

| 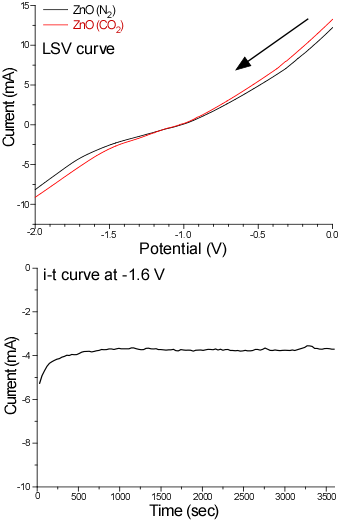 | 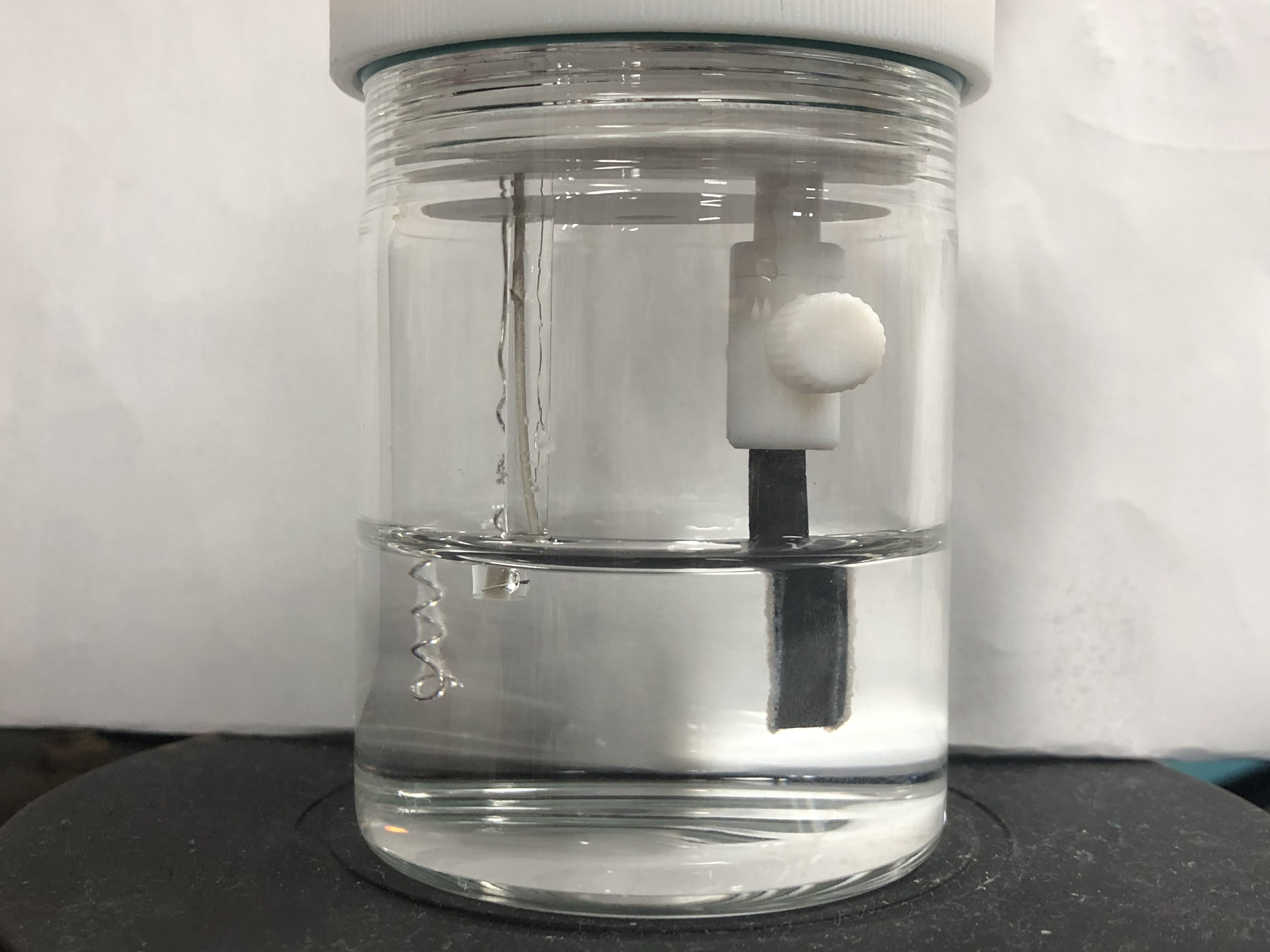 |
| --- | --- |

**Fig. S2.** Typical LSV (left top) and amperometry (left bottom) curves, and the photo (right) of the cell taken during the LSV. As seen in the photo, as the potential was changed from zero to -2.0 V ZnO became a soluble product and milky-type materials were fallen from the electrode. During an amperometry experiment**,** we did not observe this phenomenon and the current was observed to be fairly stable with time. **Table S1**. Electrochemical CO_2_ reduction ppm amounts and FE(%) for ZnO_NR_, In(240s)/ZnO_NR_, and GZO/In(240s)/ZnO_NR_ with GZO thicknesses of 10 s, 100 s, and 800 s at −1.6 V (vs. Ag/AgCl) for 1 h displayed in Figure 3.

| ppm | H_2_ | CO | CH_4_ | C_2_H_2_ | CO/H_2_ |
| --- | --- | --- | --- | --- | --- |
| ZnO_NR_ | 1284.6 | 2178.8 | 1.5 | - | 1.7 |
| In(240s)/ZnO_NR_ | 543.7 | 3933.4 | 1.6 | 16.7 | 7.2 |
| GZO(10s)/In(240s)/ZnO_NR_ | 707 | 2108.2 | 1.5 | - | 3.0 |
| GZO(100s)/In(240s)/ZnO_NR_ | 779.6 | 2381.6 | 1.4 | 4.1 | 3.1 |
| GZO(800s)/In(240s)/ZnO_NR_ | 1445.7 | 3675.3 | 1.6 | - | 2.5 |

| FE (%) | H_2_ | CO | CH_4_ | C_2_H_2_ | acetone | formate |
| --- | --- | --- | --- | --- | --- | --- |
| ZnO_NR_ | 9.33 | 15.83 | 0.04 | - | - | - |
| In(240s)/ZnO_NR_ | 2.07 | 15.01 | 0.02 | 0.32 | 0.35 | 7.52 |
| GZO(10s)/In(240s)/ZnO_NR_ | 1.58 | 4.72 | 0.01 |  |  | 3.28 |
| GZO(100s)/In(240s)/ZnO_NR_ | 4.47 | 13.64 | 0.03 | 0.12 | 0.13 | 6.17 |
| GZO(800s)/In(240s)/ZnO_NR_ | 6.11 | 15.52 | 0.03 | - | - | 4.40 |

**Table S2**. Electrochemical CO_2_ reduction ppm amounts and FE(%) for ZnO_NR_, GZO(100s)/ZnO_NR_, GZO(100s)/In/ZnO_NR_ with In thicknesses of 60 s, 240 s, and 960 s at −1.6 V (vs. Ag/AgCl) for 1 h, displayed in Figure 3.

| ppm | H_2_ | CO | CH_4_ | C_2_H_2_ | CO/H_2_ |
| --- | --- | --- | --- | --- | --- |
| ZnO_NR_ | 1284.6 | 2178.8 | 1.5 | - | 1.7 |
| GZO(100s)/ZnO_NR_ | 696.4 | 3689 | 1.9 | - | 5.3 |
| GZO(100s)/In(60s)/ZnO_NR_ | 1464.5 | 1966.1 | 1.5 | - | 1.3 |
| GZO(100s)/In(240s)/ZnO_NR_ | 779.6 | 2381.6 | 1.4 | 4.1 | 3.1 |
| GZO(100s)/In(960s)/ZnO_NR_ | 495.8 | 1826.7 | 1.3 | 2.2 | 3.7 |

| FE (%) | H_2_ | CO | CH_4_ | C_2_H_2_ | acetone | formate |
| --- | --- | --- | --- | --- | --- | --- |
| ZnO_NR_ | 9.33 | 15.83 | 0.04 | - | - | - |
| GZO(100s)/ZnO_NR_ | 3.01 | 15.95 | 0.03 | - | 0.20 | 2.80 |
| GZO(100s)/In(60s)/ZnO_NR_ | 10.60 | 14.23 | 0.04 | - | - | 4.72 |
| GZO(100s)/In(240s)/ZnO_NR_ | 4.47 | 13.64 | 0.03 | 0.12 | 0.13 | 6.17 |
| GZO(100s)/In(960s)/ZnO _NR_ | 3.81 | 14.02 | 0.04 | 0.08 | - | 25.80 |

**Table S3**. Errors of gaseous H_2_ and CO products

| ppm | H_2_ | CO |
| --- | --- | --- |
| ZnO_NR_ | 7.4 ± 1.9 | 18.2 ± 2.4 |
| In(240)ZnO_NR_ | 1.4 ± 0.7 | 15.5 ± 0.5 |
| GZO(10s)/In(240s)/ZnO_NR_ | 1.5 ± 0.0 | 12.2 ± 7.5 |
| GZO(100s)/In(240s)/ZnO_NR_ | 2.8 ± 1.7 | 14.5 ± 0.8 |
| GZO(800s)/In(240s)/ZnO_NR_ | 5.6 ± 0.5 | 16.3 ± 0.8 |

| ppm | H_2_ | CO |
| --- | --- | --- |
| ZnO_NR_ | 7.4 ± 1.9 | 18.2 ± 2.4 |
| GZO(100s)/ZnO_NR_ | 4.0 ± 1.0 | 17.2 ± 1.2 |
| GZO(100s)/In(60s)/ZnO_NR_ | 7.2 ± 3.4 | 18.0 ± 3.7 |
| GZO(100s)/In(240s)/ZnO_NR_ | 3.2 ± 1.2 | 18.0 ± 4.4 |
| GZO(100s)/In(960s)/ZnO_NR_ | 2.9 ± 0.9 | 18.1 ± 4.0 |

**Table S4**. Electrochemical CO_2_ reduction ppm amounts and FE(%) for GZO(100s)/In(960s)/ZnO_NR_ at different applied potentials of −1.2 V, −1.4 V, −1.6 V, and −1.8 V (vs. Ag/AgCl) for 1 h, displayed in Figure 4.

| ppm | H_2_ | CO | CH_4_ | C_2_H_2_ | CO/H_2_ |
| --- | --- | --- | --- | --- | --- |
| −1.2 V | 202.1 | 147.6 | 1.5 | 3.8 | 0.7 |
| −1.4 V | 358.5 | 1829.9 | 1.8 | 3.9 | 5.1 |
| −1.6 V | 495.8 | 1826.7 | 1.3 | - | 3.7 |
| −1.8 V | 2676.4 | 5827.1 | 1.4 | 5.7 | 2.2 |

| FE (%) | H_2_ | CO | CH_4_ | C_2_H_2_ | acetone | formate |
| --- | --- | --- | --- | --- | --- | --- |
| −1.2 V | 7.16 | 5.23 | 0.21 | 0.67 | - | - |
| −1.4 V | 2.91 | 14.86 | 0.06 | 0.16 | - | 5.30 |
| −1.6 V | 3.81 | 14.02 | 0.04 | - | - | 25.80 |
| −1.8 V | 5.16 | 11.23 | 0.01 | 0.05 | - | 22.90 |

**Table S5**. Electrochemical CO_2_ reduction ppm amounts and FE(%) for GZO(100s)/In(960s)/ZnO_NR_ electrode at −1.6 V (vs. Ag/AgCl) for 1 h under dark and 365 nm light conditions, displayed in Figure 5.

| ppm | H_2_ | CO | CH_4_ | C_2_H_2_ | CO/H_2_ |
| --- | --- | --- | --- | --- | --- |
| dark | 495.8 | 1826.7 | 1.3 | - | 3.7 |
| 365nm ON | 961 | 9873.7 | 4.9 | 4.8 | 10.3 |

| FE(%) | H_2_ | CO | CH_4_ | C_2_H_2_ | acetone | formate |
| --- | --- | --- | --- | --- | --- | --- |
| dark | 3.81 | 14.02 | 0.04 | - | - | 25.80 |
| 365nm ON | 2.65 | 27.28 | 0.05 | 0.07 | - | 4.52 |


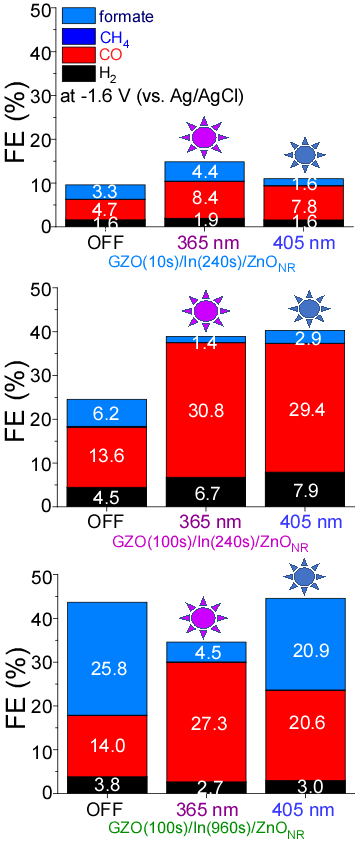


**Fig. S3.** Electrochemical CO_2_ reduction ppm amounts and FE(%) for GZO(10s)/In(240s)/ZnO_NR_, GZO(100s)/In(240s)/ZnO_NR_, and GZO(100s)/In(960s)/ZnO_NR_ electrodes at −1.6 V (vs. Ag/AgCl) for 1 h under dark, 365 nm, and 405 nm light conditions.


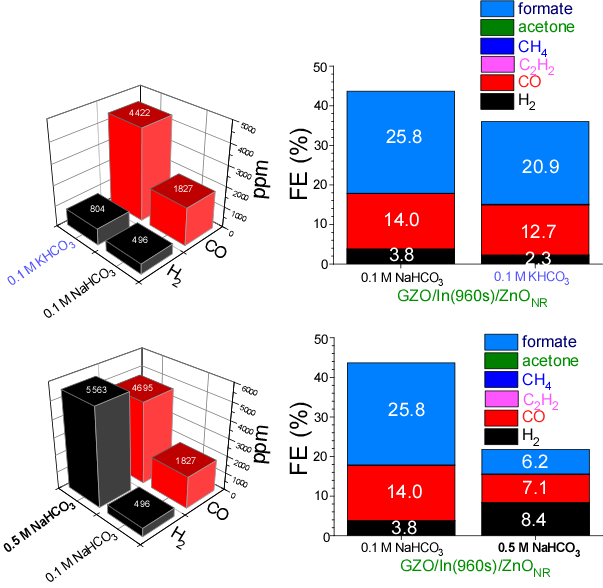


**Fig. S4**. Electrochemical CO_2_ reduction product amounts (ppm) and FE(%) for GZO(100s)/In(960s)/ZnO_NR_ electrode at −1.6 V (vs. Ag/AgCl) for 1 h in 0.1 M NaHCO_3_ and 0.1 M KHCO_3_, and 0.5 M NaHCO_3_ electrolyte conditions.

**Table S6**. Electrochemical CO_2_ reduction product amounts (ppm) and FE(%) for GZO(100s)/In(960s)/ZnO_NR_ electrode at −1.6 V (vs. Ag/AgCl) for 1 h in 0.1 M NaHCO_3_ and 0.1 M KHCO_3_, and 0.5 M NaHCO_3_ electrolyte conditions, displayed in Fig. S3.

| ppm | H_2_ | CO | CH_4_ | C_2_H_2_ | CO/H_2_ |
| --- | --- | --- | --- | --- | --- |
| 0.1 M NaHCO_3_ | 495.8 | 1826.7 | 1.3 | - | 3.7 |
| 0.1 M KHCO_3_ | 803.7 | 4422.1 | 1.4 | 0.4 | 5.5 |
| 0.5 M NaHCO_3_ | 5562.99 | 4694.9 | 1.7 | 12 | 0.8 |

| FE(%) | H_2_ | CO | CH_4_ | C_2_H_2_ | acetone | formate |
| --- | --- | --- | --- | --- | --- | --- |
| 0.1 M NaHCO_3_ | 3.81 | 14.02 | 0.04 | - | - | 25.8 |
| 0.1 M KHCO_3_ | 2.3 | 12.67 | 0.02 | 0.01 | 0.09 | 20.9 |
| 0.5 M NaHCO_3_ | 8.41 | 7.1 | 0.01 | 0.09 | - | 6.2 |

**Table S7**. CO_2_ reduction performances of Zn-based catalysts in the literatures.

| Electrode | Electrolyte | Operating potential | Current density  (mA/cm^2^) | Faradaic efficiency | Reference |
| --- | --- | --- | --- | --- | --- |
| Zn-nanoparticles | 0.1 M NaHCO_3_ | −1.1 V (vs. RHE) | 5 | ~65% (CO)  ~25% (H_2_)  ~10% (HCOOH) | [1] |
| Zn porous network | 0.5 M KHCO_3_ | −1.1 V (vs RHE) | ~16 | ~80% (CO)  ~10% (HCOOH)  ~10% (H_2_) | [2] |
| Zn nanosheets | 0.1 M KHCO_3_ | −1.0 V (vs. RHE) | ~8 | 90% (CO)  ~10% (H_2_) | [3] |
| Multilayered Zn nanosheets | 0.5 M NaHCO_3_ | −1.13 V (vs. RHE) | 5.2 | 86% (CO)  - | [4] |
| oxide-derived ZnO | 1 M KOH | -0.62V (vs. RHE) | 200 | 91.6% (CO)  ~8% (H_2_) | [5] |
| ZnO with oxygen vacancy | 0.1 M KHCO_3_ | −1.1 V (vs. RHE) | ~19 | 83% (CO) | [6] |
| Porous ZnO Nanosheets with Hydroxyl Groups | ([Bmim]PF_6_, 30 wt%)/acetonitrile/H_2_O (5 wt%) | -2.0 V (vs. Ag/AgCl) | 44.3 | 97.8% (CO)  ~2% (H_2_) | [7] |
| In/ZnO@C hollow nanocubes | 0.5 M KHCO_3_ | −1.2 V (vs. RHE) | 23.5 | 90% (HCOO^-^)  8% (H_2_)  2% (CO) | [8] |
| indium–zinc bimetallic  nanocrystals | 0.5 M KHCO_3_ | -1.2 V (vs. RHE) | -22 | 95% (HCOOH)  ~5% (H_2_, CO) | [9] |
| SnO_2_/ZnO Composite Hollow Nanofiber | 0.5 M KHCO_3_ | -1.34 V (vs. RHE) | 24.9 | 97.9 % (HCOO^-^)  ~2% (CO,H_2_) | [10] |
| low-crystallinity mesoporous ZnGa_2_O_4_ | 0.1 M KHCO_3_ | -1.4 V (vs. Ag/AgCl) | 0.5 | 96 % (CO)  ~4% (H_2_) | [11] |
| sandwiched hybrid Ga_2_O_3_:ZnO/Indium/ZnO nanorods | 0.1 M NaHCO_3_ | -1.6 V (vs. Ag/AgCl) | 5 | 26% (HCOO^-^)  14%(CO)  4%(H_2_) | This work |

[1] H.S. Jeon, I. Sinev, F. Scholten, N.J. Divins, I. Zegkinoglou, L. Pielsticker, B.R. Cuenya, Operando Evolution of the Structure and Oxidation State of Size-Controlled Zn Nanoparticles during CO_2_ Electroreduction, J. Am. Chem. Soc. 140 (2018) 9383–9386. doi:10.1021/jacs.8b05258.

[2] Y. Lu, B. Han, C. Tian, J. Wu, D. Geng, D. Wang, Efficient electrocatalytic reduction of CO_2_ to CO on an electrodeposited Zn porous network, Electrochem. Commun. 97 (2018) 87-90. doi:10.1016/j.elecom.2018.11.002

[3] K. Liu, J. Wang, M. Shi, J. Yan, Q. Jiang, Simultaneous Achieving of High Faradaic Efficiency and CO Partial Current Density for CO_2_ Reduction via Robust, Noble-Metal-Free Zn Nanosheets with Favorable Adsorption Energy, Adv. Energy Mater. 9 (2019) 1900276. doi:10.1002/aenm.201900276.

[4] T. Zhang, X. Li, Y. Qiu, P. Su, W. Xu, H. Zhong, H. Zhang, Multilayered Zn nanosheets as an electrocatalyst for efficient electrochemical reduction of CO_2_, J. Catal. 357 (2018) 154-162. doi: 10.1016/j.jcat.2017.11.003

[5] W. Luo, Q. Zhang, J. Zhang, E. Moioli, K. Zhao, A. Züttel, Electrochemical reconstruction of ZnO for selective reduction of CO_2_ to CO. Appl. Catal. B: Environ. 273 (2020) 119060. doi:10.1016/j.apcatb.2020.119060

[6] Z. Geng, X. Kong, W. Chen, H. Su, Y. Liu, F. Cai, G. Wang, J. Zeng, Oxygen Vacancies in ZnO Nanosheets Enhance CO_2_ Electrochemical Reduction to CO, Angew. Chemie - Int. Ed. 57 (2018) 6054–6059. doi:10.1002/anie.201711255.

[7] H. Wang, D. Yang, J. Yang, X. Ma, H. Li, W. Dong, R. Zhang, C. Feng, Efficient electroreduction of CO_2_ to CO on porous ZnO nanosheets with hydroxyl groups in ionic liquid-based electrolytes, ChemCatChem 13 (2021) 2570-2576. doi: 10.1002/cctc.202100329

[8] X. Teng, Y. Niu, S. Gong, M. Xu, X. Liu, L. Ji, Z. Chen, In/ZnO@ C hollow nanocubes for efficient electrochemical reduction of CO 2 to formate and rechargeable Zn–CO 2 batteries. Mater. Chem. Front. 5 (2021) 6618-6627. doi:10.1039/D1QM00825K

[9] I. S. Kwon, T. T. Debela, I. H. Kwak, H. W. Seo, K. Park, D. Kim, S. J. Yoo, J. K. Kim, J. Park, H. S. Kang, Selective electrochemical reduction of carbon dioxide to formic acid using indium–zinc bimetallic nanocrystals. J. Mater. Chem. A 7 (2019) 22879-22883. doi: 10.1039/c9ta06285h

[10] D. Tan, W. Lee, Y.E. Kim, Y.N. Ko, M.H. Youn, Y.E. Jeon, J. Hong, S.K. Jeong, K.T. Park, SnO_2_/ZnO composite hollow nanofiber electrocatalyst for efficient CO_2_ reduction to formate, ACS Sustain. Chem. Eng. 8 (2020) 10639-10645. doi: 10.1021/acssuschemeng.0c03481

[11] M. Zhao, Y. Gu, P. Chen, Z. Xin, H. Zhu, B. Wang, K. Zhu, S. Yan, Z. Zou, Highly selective electrochemical CO_2_ reduction to CO using a redox-active couple on low-crystallinity mesoporous ZnGa_2_O_4_ catalyst, J. Mater. Chem. A 7 (2019) 9316-9323. doi: 10.1039/C9TA00562E
